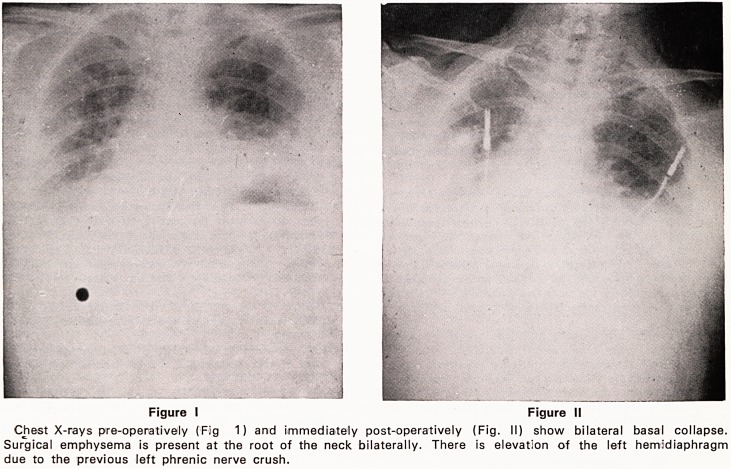# An Atypical Case of Spontaneous Rupture of the Oesophagus

**Published:** 1975-01

**Authors:** A. A. J. Barros D'Sa

**Affiliations:** Musgrove Park Hospital, Taunton, Somerset

## Abstract

A case of spontaneous rupture of the oesophagus is described in which the presentation is unusual. The rarity of the condition is emphasized. The diagnosis and treatment are briefly considered and the difficulties in diagnosis stressed. The similarities and differences between this condition and the Mallory-Weiss syndrome are discussed.


### An Atypical case of Spontaneous Rupture of the Oesophagus

A. A. J. Barros D'Sa, M.B., Ch.B., M.R.C.S., L.R.C.P., F.R.C.S.E.

Musgrove Park Hospital, Taunton, Somerset

*Present address: Department of Surgery, University Hospital of Wales, The Heath, Cardiff.

**Summary**

A case of spontaneous rupture of the oesophagus is described in which the presentation is unusual. The rarity of the condition is emphasized. The diagnosis and treatment are briefly considered and the difficulties in diagnosis stressed. The similarities and differences between this condition and the Mallory-Weiss syndrome are discussed.

**Case Report**

Early one morning a 50 year old factory worker was admitted to hospital with abdominal pain. He gave a history of "indigestion for years", flatulence after meals, epigastric pain about two hours after meals, and of being woken up in the middle of the night by this pain. The pain was relieved by the taking of food, milk or Rennies. He had a left phrenic crush for pulmonary tuberculosis twenty years previously.

The previous evening he had an episode of vomiting and some abdominal pain. The pain was only a dull ache at first. It was not until several hours later that it became severe and felt like a knife sticking into his abdomen. There had been no blood in his vomitus.

Clinically he was groaning in pain, his respirations were shallow and his pulse was over 130 per minute. His temperature was normal and there were no abnormal sounds on auscultation of his chest. The whole of his abdomen was rigid and silent. A provisional diagnosis of a perforated peptic ulcer was made and it was not until after the patient had returned from the X-ray department that a small area of subcutaneous emphysema was found in each supra-clavicular fossa. This was about 10 hours after his first symptom. The emphysema very soon spread to involve both sides of his neck (Figures I and II) and it was now clear that he had ruptured his oesophagus.

Figure I

Figure II

Chest X-rays pre-operatively (Fig 1) and immediately post-operatively (Fig. II) show bilateral basal collapse. Surgical emphysema is present at the root of the neck bilaterally. There is elevation of the left hemidiaphragm due to the previous left phrenic nerve crush.

At operation, through a left thoracotomy incision, a great deal of purulent fluid and bits of food were removed from the pleural cavity. A longitudinal tear, 3 cms long, on the antero-lateral aspect of the lower end of the oesophagus was repaired and the chest drained by under-water seal. Another drain in the right pleural cavity drained a large accumulation of purulent fluid.

The patient was put on a large dose of penicillin together with streptomycin and hydrocortisone. After an uneventful post-operative recovery he was discharged home 20 days after his operation. A year after his operation he was perfectly well, and a barium swallow showed a smooth normal oesophagus, and two years later he was still asymptomatic.

**Discussion**

Boerhaave (1724) first described a case of spontaneously occurring tear in the lower oesophagus with a fatal outcome from mediastinitis. Spontaneous rupture of the non-diseased oesophagus is a rare occurrence but one which is invariably fatal unless promptly recognised and adquately treated (Callaghan, 1972).

One of the first symptoms of spontaneous rupture is agonizing pain in the chest, preceded by violent vomiting or retching. The pain is more intense than that of a perforated gastric ulcer (d'Abreu, 1965). This initial symptom was absolutely atypical in this patient. Not only was the pain described as a "dull ache", it did not become severe until several hours had elapsed. In fact the pain did not worry him particularly at first and was certainly much less severe than the epigastric pain he used to suffer after meals. It is true that he had vomited a little but this was by no means violent or prolonged.

The diagnostic signs of this condition are rapid shallow respirations, upper abdominal rigidity and subcutaneous emphysema. The rigidity is classically restricted to the upper abdomen, but rigidity of all four quadrants of the abdomen as in this case has been reported (Tidman and John, 1967; Roberts and Messent, 1967). In 95% of cases the characteristic site of the tear is the left postero-lateral aspect of the distal oesophagus (Callaghan, 1972). However, a lipiodol or gastrograffin swallow is always valuable before operation, not only because the leak can be into both pleural cavities but because the rupture may be higher up in the oesophagus.

Treatment, except in late cases where intercostal drainage is used, is surgical repair with closed drainage of the chest. Until 1946 fifty definite cases were recorded in the literature but none had survived (Barrett, 1946). The first successful repair was carried out in 1946 (Barrett, 1947). It is now generally agreed that the best treatment is surgical repair as soon as possible, except in the very late case with a fulminating type of emphysema where intercostal drainage is necessary.

This condition can easily be misdiagnosed as perforated peptic ulcer, myocardial infarction, dissecting aneurysm of the aorta, spontaneous pneumothorax or acute pancreatitis. There are two important reasons for a misdiagnosis. The first is its rarity. This is the first case that is ever known to have been admitted to this large District Hospital which has a catchment population of 250,000. Even in a large thoracic unit at Frenchay Hospital, Bristol, Belsey sees no more than about one case of spontaneous rupture of the oesophagus a year (Belsey, 1974). The second reason for a misdiagnosis is if the patient has, as this one did, a definite history of peptic ulceration. Even in the absence of a peptic ulcer history, perforated peptic ulcer is the most common misdiagnosis (Ware et al., 1952).

Differentiation of rupture of the oesophagus from the Mallory-Weiss syndrome is essential. In this syndrome the mucosal tear is superficial and does not extend through the muscular layer. In both cases the tears are longitudinal, but while the complete rupture is restricted to the oesophagus the mucosal tear in the Mallory-Weiss syndrome extends from the cardia upwards into the oesophagus and downwards into the stomach. Spontaneous rupture is usually preceded by excessive retching or vomiting and associated with severe retrosternal pain. In contrast, the main features of a mucosal tear are those associated with blood loss and there may be little or no pain (Smith et al., 1974). Radiography is usually of no value in the Mallory-Weiss syndrome and conservative management is sufficient. There is no doubt that radiography is of great value in the diagnosis of complete rupture and operative intervention is essential here.

**Acknowledgements**

I would like to thank Mr. R. D. Rowlands for allowing me to operate on this patient and for permission to publish this case report of a patient that was under his care.

**REFERENCES**

BARRETT, N. R. Spontaneous perforation of the oesophagus. 1946 **Thorax, 1,** 48-70.

BARRETT, N. R. Report of a case of spontaneous perforation of the oesophagus successfully treated by operation. **British Journal of Surgery,** 1947, **35,** 216-218.

BELSEY, 1974. Personal communication.

BOERHAAVE, H. (1724) Atrocis nec descripti prius, morbi hostoria secundum medicae artis leges conscripta. Cited by Moynihan, N. H. **Lancet,** 1954, **2,** 728-731.

CALLAGHAN, J. The Boerhaave Syndrome (spontaneous rupture of the oesophagus). **British Journal of Surgery,** 1972, **59,** 41-44.

d'ABREU, A. L. Spontaneous rupture of the oesophagus. In: **Clinical Surgery** 5 Thorax, Rob, C. and Smith, R. London: Butterworth, 1965, pp. 341-346.

ROBERTS, D. M. AND MESSENT, D. O. H. Spontaneous rupture of the oesophagus with unusual features. **Postgraduate Medical Journal,** 1967, **43,** 614-618.

SMITH, G., BRUNNEN, P. L., GILLANDERS, L. A., TEO, H. S. Oesophageal apoplexy. **Lancet,** 1974, **1,** 390-392.

TIDMAN, M. K. AND JOHN, H. T. Spontaneous rupture of the oesophagus. **British Journal of Surgery,** 1967, **54,** 286-292.

WARE, G. W., SHNIDER, B. I. AND DAVIS, E. W. Spontaneous rupture of the oesophagus. **Archives of Surgery,** 1952, **65,** 723-745.

## Figures and Tables

**Figure I Figure II f1:**